# Hypoxia Inducible Factor 1-Alpha (HIF-1 Alpha) Is Induced during Reperfusion after Renal Ischemia and Is Critical for Proximal Tubule Cell Survival

**DOI:** 10.1371/journal.pone.0033258

**Published:** 2012-03-14

**Authors:** Elisa Conde, Laura Alegre, Ignacio Blanco-Sánchez, David Sáenz-Morales, Elia Aguado-Fraile, Belén Ponte, Edurne Ramos, Ana Sáiz, Carlos Jiménez, Angel Ordoñez, Manuel López-Cabrera, Luis del Peso, Manuel O. de Landázuri, Fernando Liaño, Rafael Selgas, Jose Antonio Sanchez-Tomero, María Laura García-Bermejo

**Affiliations:** 1 Department of System Disorders and Cancer, Instituto Ramón y Cajal de Investigación Sanitaria (IRYCIS), Alcalá University, Madrid, Spain; 2 Department of Nephrology, Instituto de Investigación La Princesa (IP), Madrid, Spain; 3 Department of Nephrology, Hospital La Paz (IdIPaz), Madrid, Spain; 4 Department of Immunology, Instituto de Investigación La Princesa (IP), Madrid, Spain; 5 Centro de Biología Molecular Severo Ochoa, CSIC-UAM, Cantoblanco, Madrid, Spain; 6 HIV Unit, Department of Biochemistry, Hospital La Paz (IdiPAZ), Autónoma University School of Medicine, Institute of Biomedical Research Alberto Sols, CSIC-UAM, Madrid, Spain; 7 Department of Nephrology, Hospital Ramón y Cajal, Madrid, Spain; 8 Instituto Ramón y Cajal de Investigación Sanitaria (IRYCIS), Alcalá University, Madrid, Spain; University of Sao Paulo Medical School, Brazil

## Abstract

Acute tubular necrosis (ATN) caused by ischemia/reperfusion (I/R) during renal transplantation delays allograft function. Identification of factors that mediate protection and/or epithelium recovery could help to improve graft outcome. We studied the expression, regulation and role of hypoxia inducible factor 1-alpha (HIF-1 α), using *in vitro* and *in vivo* experimental models of I/R as well as human post-transplant renal biopsies. We found that HIF-1 α is stabilized in proximal tubule cells during ischemia and unexpectedly in late reperfusion, when oxygen tension is normal. Both inductions lead to gene expression *in vitro* and *in vivo*. *In vitro* interference of HIF-1 α promoted cell death and *in vivo* interference exacerbated tissue damage and renal dysfunction. In pos-transplant human biopsies, HIF-1 α was expressed only in proximal tubules which exhibited normal renal structure with a significant negative correlation with ATN grade. In summary, using experimental models and human biopsies, we identified a novel HIF-1 α induction during reperfusion with a potential critical role in renal transplant.

## Introduction

Ischemia is one of the most frequent causes of acute renal failure [1], chronic kidney disease and also occurs during kidney transplantation. Indeed, development of ATN significantly contributes to renal allograft function delay [2]. In spite of the advances in the immunosuppressive therapy, there is little improvement in ATN recovery during renal transplant.

HIF-1 α is the master regulator of cell response to hypoxia since it leads to the expression of several genes involved in adaptation to decreased oxygen availability [3]. HIF is a heterodimeric protein including an oxygen-regulated alpha subunit and a constitutive expressed beta subunit. Alpha subunits are degraded during normoxia mainly through a proteasome-dependent pathway after hydroxylation of two proline residues by prolyl-hydroxilases (PHDs). During hypoxia, PHDs are inhibited and HIF-1 α subunit accumulates, dimerizes with HIF-1β and drive expression of HIF target genes which include genes involved in angiogenesis and tissue repair such as vascular endothelial growth factor (VEGF) or erythropoietin (EPO) and prolilhydroxilases (PHDs) genes, among others.

Knowledge about mechanisms involved in normoxic HIF-1 α induction is just beginning to emerge. It has been shown that HIF-1 α can be up-regulated through the PI3k/Akt-mTOR pathway in response to growth factors [4], [5]. Impairment in HIF-1 α degradation can also contribute to the induction of this factor in normoxia [6]. Evidence regarding the critical role of HIF-1 α in the cell response to stimuli independently of oxygen restriction is increasing [7].

A reno-protective role of HIF against ischemic injury in I/R models [8] and toxic nephropathies has been already described [9]. Moreover, the use of PHDs inhibitors such as iron-chelators in renal transplant models indicates that HIF induction protects tubular cells from ischemic injury [10]. These investigations prove the beneficial role of HIF-1 α stabilization before or during ischemia. However, data describing non-oxygen regulated HIF-1 α expression during reperfusion after ischemia and its potential implications in renal injury outcome are scarce.

In this work, using an *in vitro* model of oxygen and nutrient alterations in the human proximal epithelial cell line HK-2 [12], an *in vivo* model of I/R [11] in rats and a set of human allograft biopsies exhibiting ATN, we have studied the expression, regulation and the potential role of HIF-1 α in the tubular response during I/R. Our results identify HIF-1 α accumulated during reperfusion as a putative target for intervention to accelerate ATN recovery after renal transplants.

## Results

### HIF-1 α is bi-phasically induced during hypoxia/reoxygenation in proximal tubule cells

**Figure 1:**
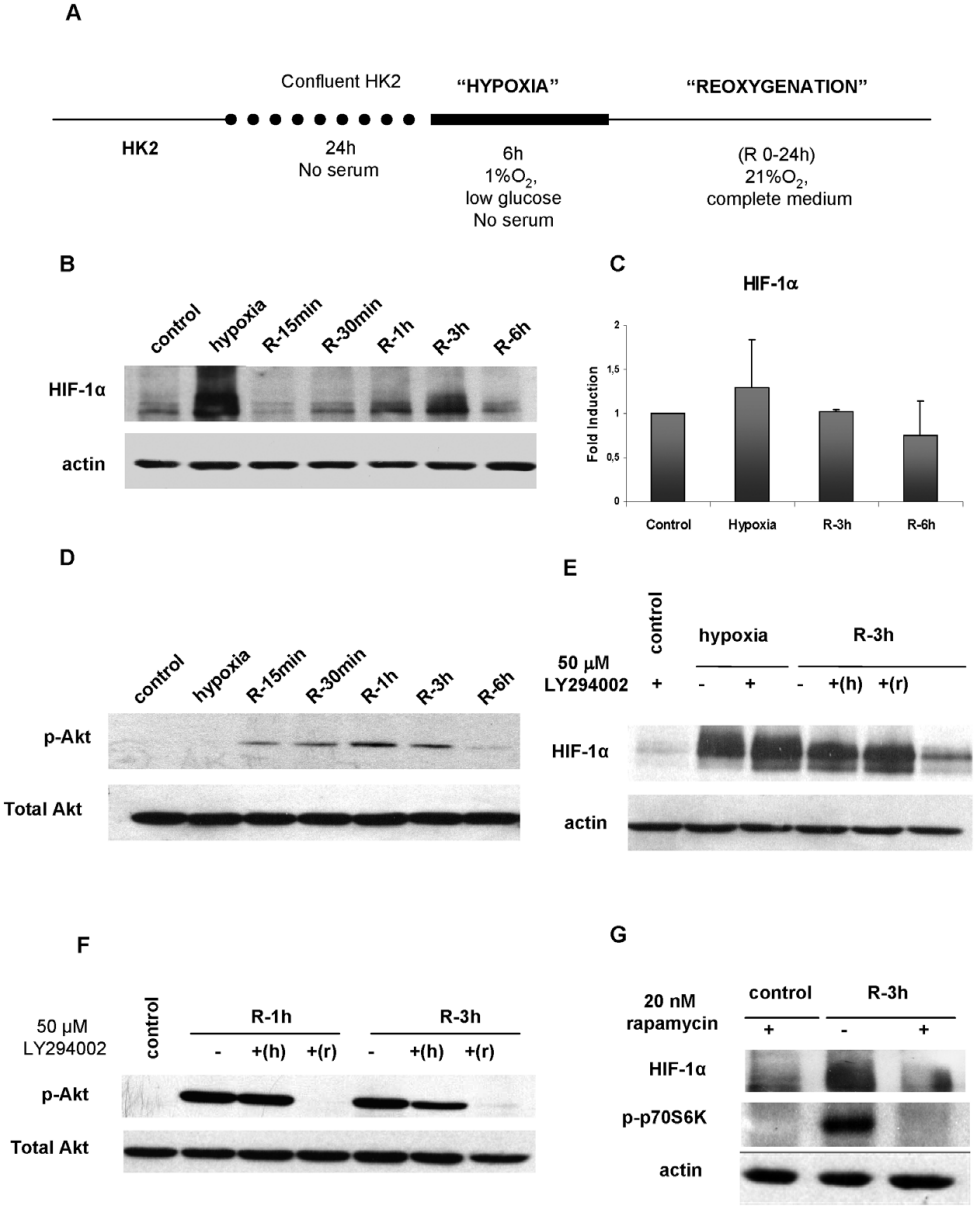
HIF-1 α expression in an *in vitro* model mimicking I/R. **Akt/mTOR signaling mediates HIF-1 α induction during reoxygenation.** (a) Scheme of *in vitro* protocol of Hypoxia/Reoxygenation (H/R) HK-2 cells were subjected to H/R protocol which includes oxygen and nutrients deprivation and replenishment. (b) HIF-1α protein expression was determined by western blot. Actin expression was used as loading control. Representative western blot are shown. (c) qRT-PCR analysis of HIF-1 α mRNA, expressed as mean ±SEM of HIF-1α levels using b-actin mRNA as internal control. No significant alterations on mRNA levels of HIF-1 α during H/R were found in four different experiments. (d) Activation of Akt estimated by western blot of 473Ser phosporylation in HK2 cells subjected to H/R. Total Akt expression was used as control. (e) Effect of LY294002 on HIF-1 α inductions: 50 mM LY294002 applied during hypoxia (+h) did not have any effect but during reoxygenation (+r) it reduced HIF-1α expression. Representative western blots are shown. (f) Control of Akt inhibition by LY294002. 50 mM of LY294002 efficiently inhibits Akt phosphorylation when added during reoxygenation (+r). (g) Effect on the HIF-1α inductions of 20 nM rapamycin applied during reoxygenation, estimated by western blot. Phosphorylation of p70S6K was used as control of rapamycin efficiency.

We had established and characterized in our laboratory an *in vitro* model of oxygen and nutrient deprivation/replenishment [11] in the human proximal epithelial cells HK-2 called hypoxia/reoxygenation (H/R) (figure 1A), which closely reproduce the stimuli and the effects of renal ischemia/reperfusion (I/R) in proximal epithelial tubule cells. Using this model, we determined HIF-1 α expression by inmunoblot (figure 1B). HIF-1 α has a biphasic pattern of induction: after hypoxia and during reoxygenation (1–3 h).

To assess whether these HIF-1 α inductions are the result of protein stabilization, qRT-PCR to estimate HIF-1 α mRNA levels was performed. No significant changes in HIF-1 α mRNA were observed (fig. 1C), indicating that in our model, HIF-1 α is regulated mainly at protein level.

These results indicate that HIF-1 α is accumulated during hypoxia but unexpectedly also during reperfusion.

### Akt/mTOR signalling is responsible for HIF-1 α accumulation during reoxygenation

Although Akt/mTOR signalling pathway is not required for hypoxia-induced HIF-1 α [13], [14], activation of Akt/mTOR pathway has been proposed as one of the mechanisms responsible for HIF-1 α stabilization in normoxia.

Therefore, we determined pAkt (Ser437) levels, by immunoblot, finding that Akt is not activated during hypoxia but is transiently activated during reoxygenation (15 min-3 h), when HIF-1 α induction is observed (figure 1D). Moreover, the use of PI3K specific inhibitor LY 294002 during reoxygenation efficiently reduced HIF-1 α expression (figure 1E), indicating that Akt activation is responsible for HIF-1 α stabilization during this period. Conversely, the use of LY did not affect HIF-1 α induction during hypoxia. LY294002 exhibited high efficiency inhibiting Akt phosphorylation (figure 1F).

Additionally, the mTOR inhibitor rapamycin was used during reoxygenation to assess mTOR implication in HIF-1 α induction (figure 1G). Inhibition of mTOR prevents HIF-1 α stabilization.

These findings demonstrate that the Akt/mTOR signalling pathway activation is responsible for HIF-1 α induction during reoxygenation, among others.

**Figure 2:**
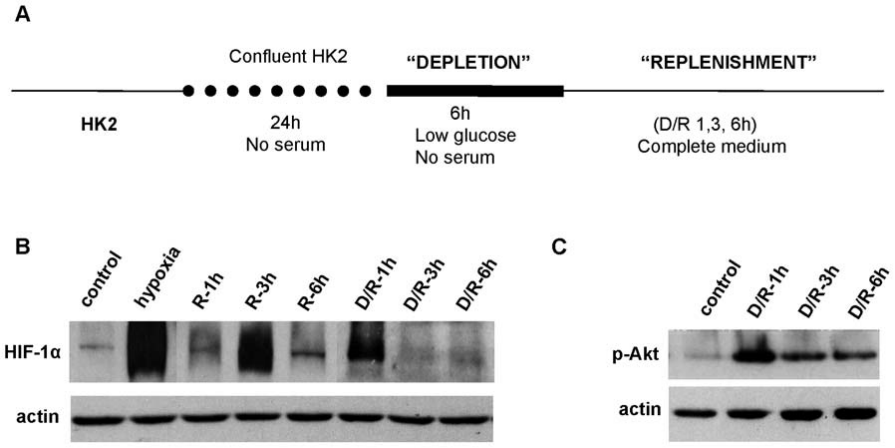
*In vitro,* HIF-1 α induction during reoxygenation is not due to low oxygen levels. (a) Scheme of *in vitro* protocol of Depletion/Replenishment (D/R). HK-2 cells were subjected to D/R which only includes nutrients deprivation and replenishment, maintaining normal oxygen tension. (b) Expression of HIF-1 α during H/R and D/R protocol, estimated by western blot. (c) Akt is also activated (p473Ser) during D/R protocol.

To determine whether HIF-1 α could accumulate independently of low oxygen levels, we set up a new protocol including changes in nutrients but not in oxygen tension, called depletion/replenishment (D/R) (figure 2A). This protocol leads exclusively to the second HIF-1 α induction although it accumulates earlier (figure 2B). Importantly Akt is also activated during D/R protocol (figure 2C). Both results demonstrate that HIF-1 α induction during reoxygenation is not due to low oxygen tension, but rather involves Akt activation.

### Both HIF-1 α inductions promote gene expression

To study whether HIF-1 α accumulations resulted in an increased activity of this factor we evaluated HIF-1 α -dependent gene expression in HK2 cells during H/R.

**Figure 3:**
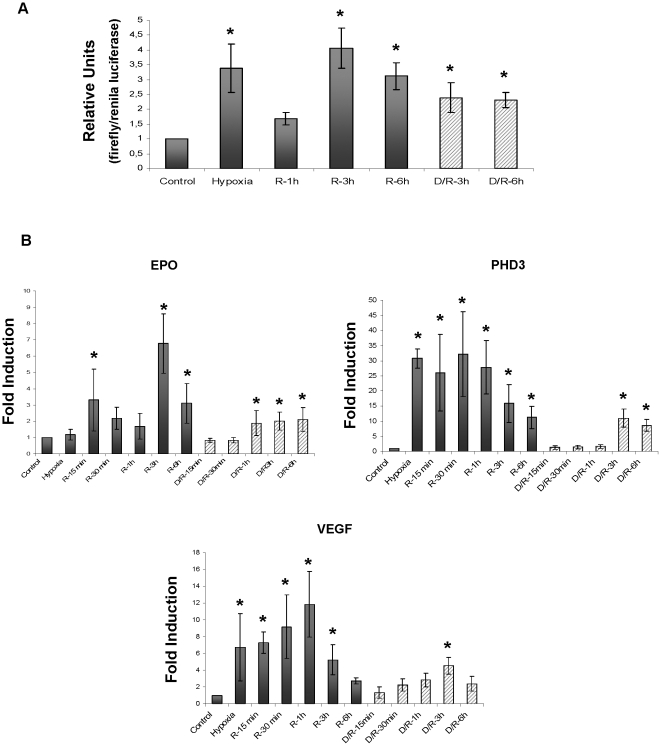
Hypoxia/reoxygenation and nutrient replenishment all induced HIF-1 α activity, promoting HIF-1 α target genes expression. (a) Luciferase reporter assays in HK2 cells subjected to H/R (grey bars) or D/R (striped bars), by transfecting 800 ng/well of 9xHRE-luc reporter and 4 ng/well of renilla-luc reporter. Data are represented as mean ±SEM of the ratio firefly/renilla luciferase of six independent experiments, relative to control condition (ratio = 1). (b) qRT-PCR to estimate mRNA expression of HIF-1α target genes EPO, PHD3, and VEGF during H/R (black bars) or D/R protocols (striped bars). Data are represented as mean ±SEM of three independent experiments, using 28 s mRNA levels as internal control. All the genes are induced during reoxygenation in both protocols. Statistical significance was found in comparison to control (H/R), p≤0.05.

We first determined the transcriptional activity of both HIF-1 α inductions by performing luciferase assays in transiently transfected HK-2 cells with the 9xHRE-luc reporter [15] (figure 3A). Both HIF-1 α inductions increase luciferase activity compared to control cells, indicating that both HIF-1 α inductions promote gene expression. D/R protocol also induced 9xHRE-luc activity, demonstrating that the normoxic HIF-1 α induction by itself promotes gene expression.

Next, we estimated the expression of HIF-1 α target genes such as EPO, VEGF and PHD3, by qRT-PCR (figure 3B). PHD3 and VEGF were markedly induced during hypoxia and both mRNAs remained elevated during reoxygenation. In contrast, EPO mRNA was expressed mainly during reoxygenation, when the second HIF-1 α induction was taking place. Interestingly and correlating with the luciferase assay results, EPO, VEGF and PHD3 mRNA were also up-regulated during D/R protocol, demonstrating that the normoxic HIF-1 α stabilization also led to gene expression.

### HIF-1 α is induced after ischemia and during reperfusion *in vivo*

To study HIF-1 α *in vivo*, we have characterized the expression pattern and activity of HIF-1 α in a model of I/R in Sprague Dawley (SD) rats. We have also assessed the oxygen availability during I/R in renal tissue using pimonidazole.

**Figure 4:**
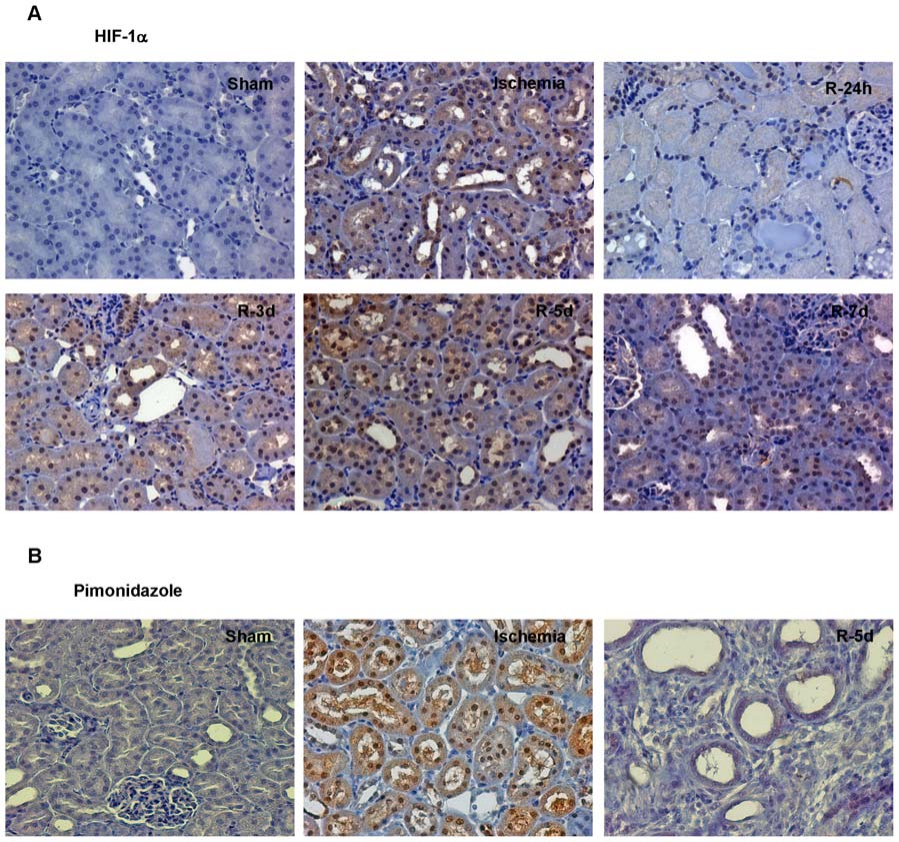
HIF-1 α is induced unexpectedly during reperfusion in rat kidney, with normal oxygen levels in renal parenchyma. (a) Immunohistochemitry to determine HIF-1 α expression in paraffin-embedded renal tissue sections from SD rats during I/R. Ischemia of 45 min and different times of reperfusion: 24 hours or 3, 5 or 7 days (R-24h, R-3d, R-5d, R-7d). HIF-1 α is detected in the nucleus of proximal tubule cells after ischemia and in reperfusion (3-5-7days). Magnification: ×400 (b) Immunostaining for pimonidazol-protein and HIF-1α adducts in renal tissue sections of rats during I/R. Ischemia of 45 min and 5 days of reperfusion. Notice positive pimonidazole immunostaining exclusively after ischemia. Magnification: ×200.

Immunohistochemistry in renal tissue demonstrated HIF-1 α expression in the nucleus of proximal tubule cells after ischemia, and, unexpectedly during reperfusion, between days 3 and 7 (figure 4A). As expected, pimonidazole staining revealed tissue hypoxia after ischemia, but it was negative at 5 days of reperfusion, indicating that tissue oxygen levels are not compromised at this moment even though HIF-1 α accumulates (figure 4B).

**Figure 5:**
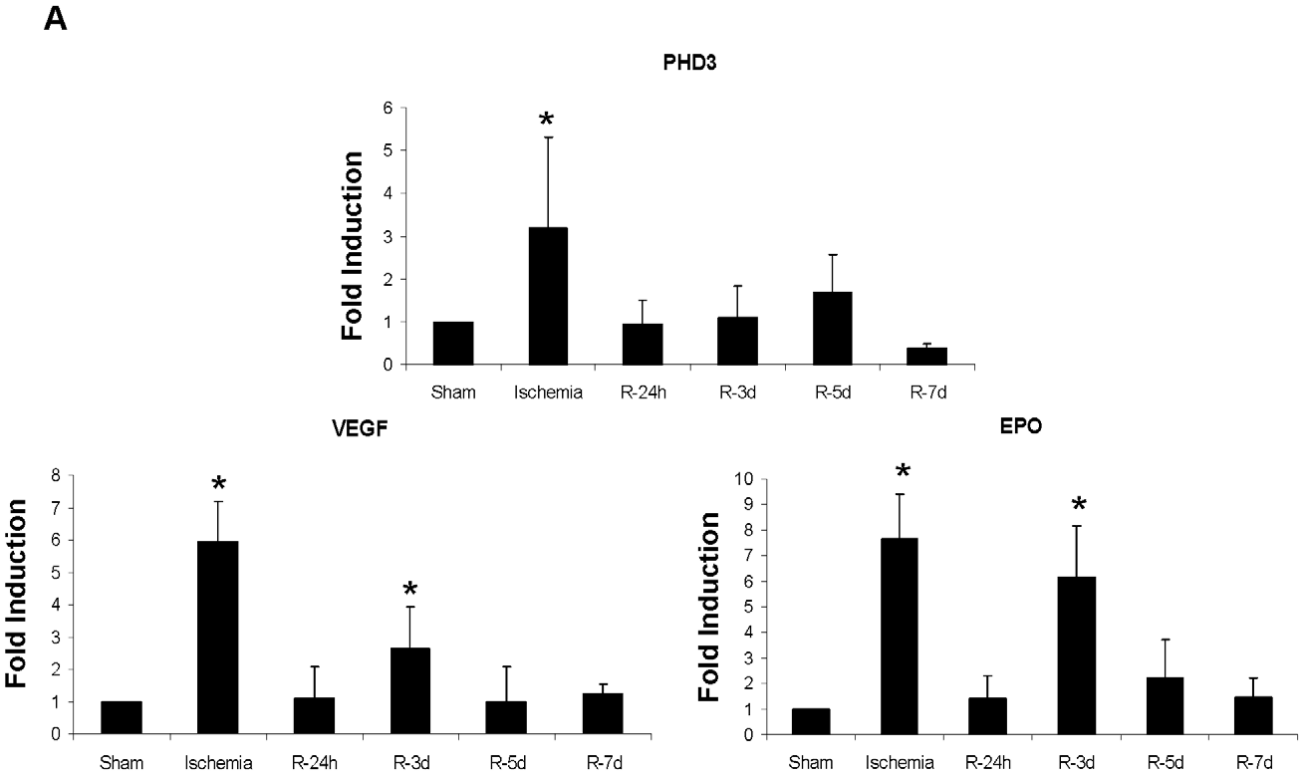
HIF-1α is transcriptionaly active *in vivo* promoting gene expression. PHD3, VEGF and EPO mRNAs levels were determined by qRT-PCR in total renal tissue lysates and 28 mRNA levels were used as internal control. Statistical significance was found in comparison to sham condition.

To determine the functionality of both HIF-1 α inductions *in vivo*, we measured the mRNA of HIF-1 α target genes including PHD3, VEGF and EPO (figure 5). All these genes are induced after ischemia and also during reperfusion. Notably, EPO mRNA is the most up-regulated during reperfusion.

These results demonstrated that HIF-1 α accumulates *in vivo* during reperfusion after renal ischemia and promotes gene expression, correlating with *in vitro* findings. The HIF-1 α induction observed during reperfusion is not due to low oxygen levels in the renal parenchyma.

### HIF-1 α mediates proximal tubule cells survival and recovery in response to I/R *in vitro* and *in vivo*

**Figure 6:**
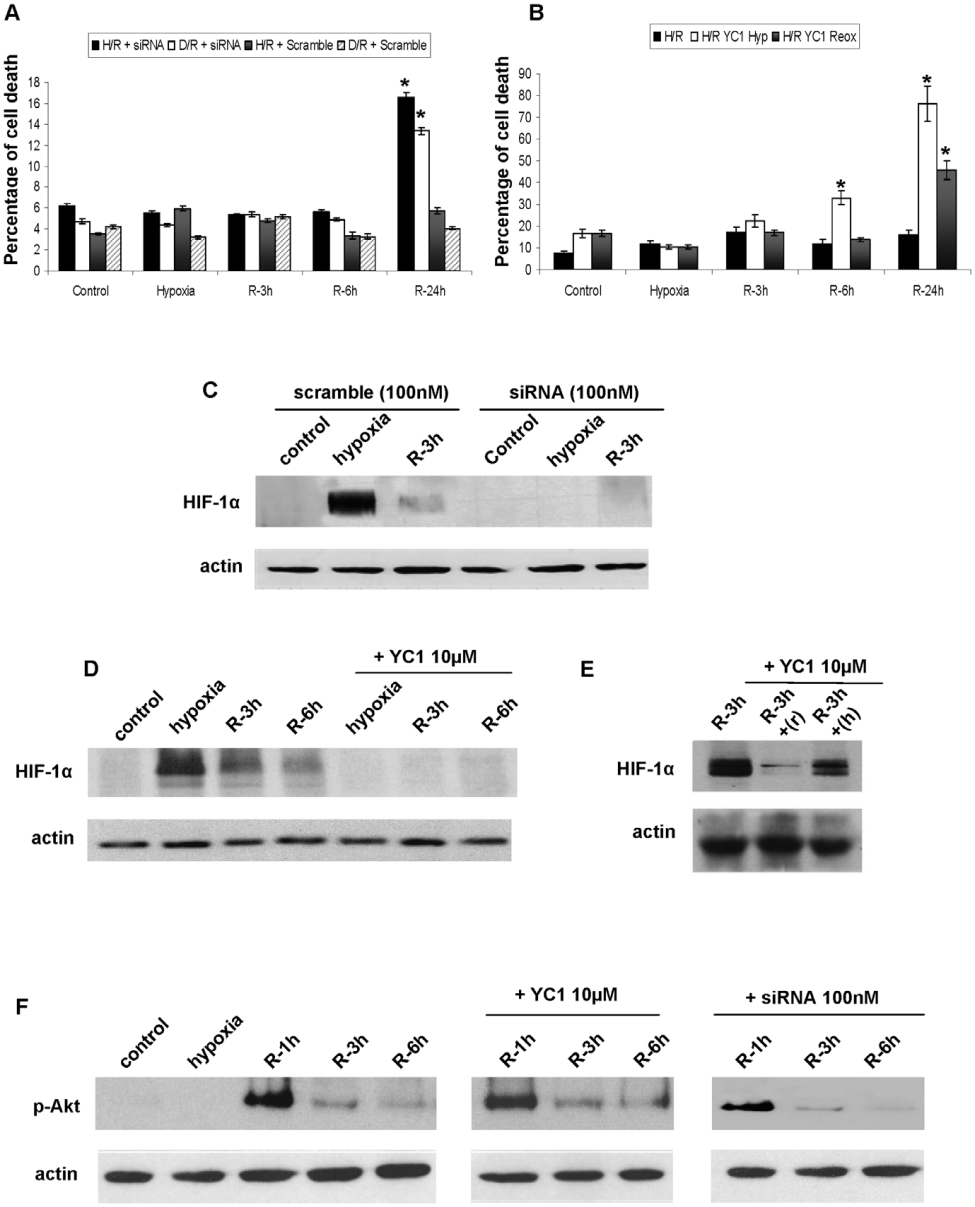
HIF-1α is required for proximal epithelial cell survival in response to oxygen and nutrient alterations. Quantification of cell death, by propidium iodide staining and flow cytometry in HK2 cells subjected to H/R and D/R, (a) previously transfected with specific HIF-1α siRNA (100 nM) and scramble (100 nM) or (b) treated with 10 mM YC-1 added during hypoxia (hyp) or during reoxygenation (reox). Statistical significance was found in comparison to scramble or control respectively, p≤0.05. (c) 100 nM of siRNA (sc-44225) efficiently prevents both HIF-1α inductions (hypoxia and R-3h). (d) 10 mM YC-1 inhibits HIF-1α inductions when added during hypoxia and reoxygenation or (e) added separately during hypoxia (+h) or reoxygenation (+r). Representative blots are shown and actin was used as control. (f) Akt activation in HK-2 cells subjected to H/R protocol, HK-2 cells treated with 10 mM YC-1 or 100 nM siRNA for HIF-1α, all estimated by western blot of 473Ser phosphorylation.

Next, we investigated the biological significance of both HIF-1 α inductions using, *in vitro*, siRNAs for HIF-1 α and YC-1, a pharmacological inhibitor of HIF-1 α. By PI staining and flow cytometry, H/R and D/R protocol did not induce cell death. The use of HIF-1 〈 siRNA (figure 6A) or YC-1 (figure 6B) in both protocols provoked significant cell death at 24 h.

Both approaches efficiently reduced HIF-1 α levels in our model (Figure 6C, 6D, 6E). YC-1 was used because siRNA does not prevent each HIF-1 α inductions separately. Notably, neither siRNA for HIF-1 α nor YC-1 affected Akt activation during reperfusion (Figure 6F).

These results demonstrated that HIF-1 α promotes proximal tubule epithelial cell survival during I/R-mimicking conditions. Outstandingly, HIF-1 α induction during reoxygenation is also critical for avoiding cell death.

**Figure 7:**
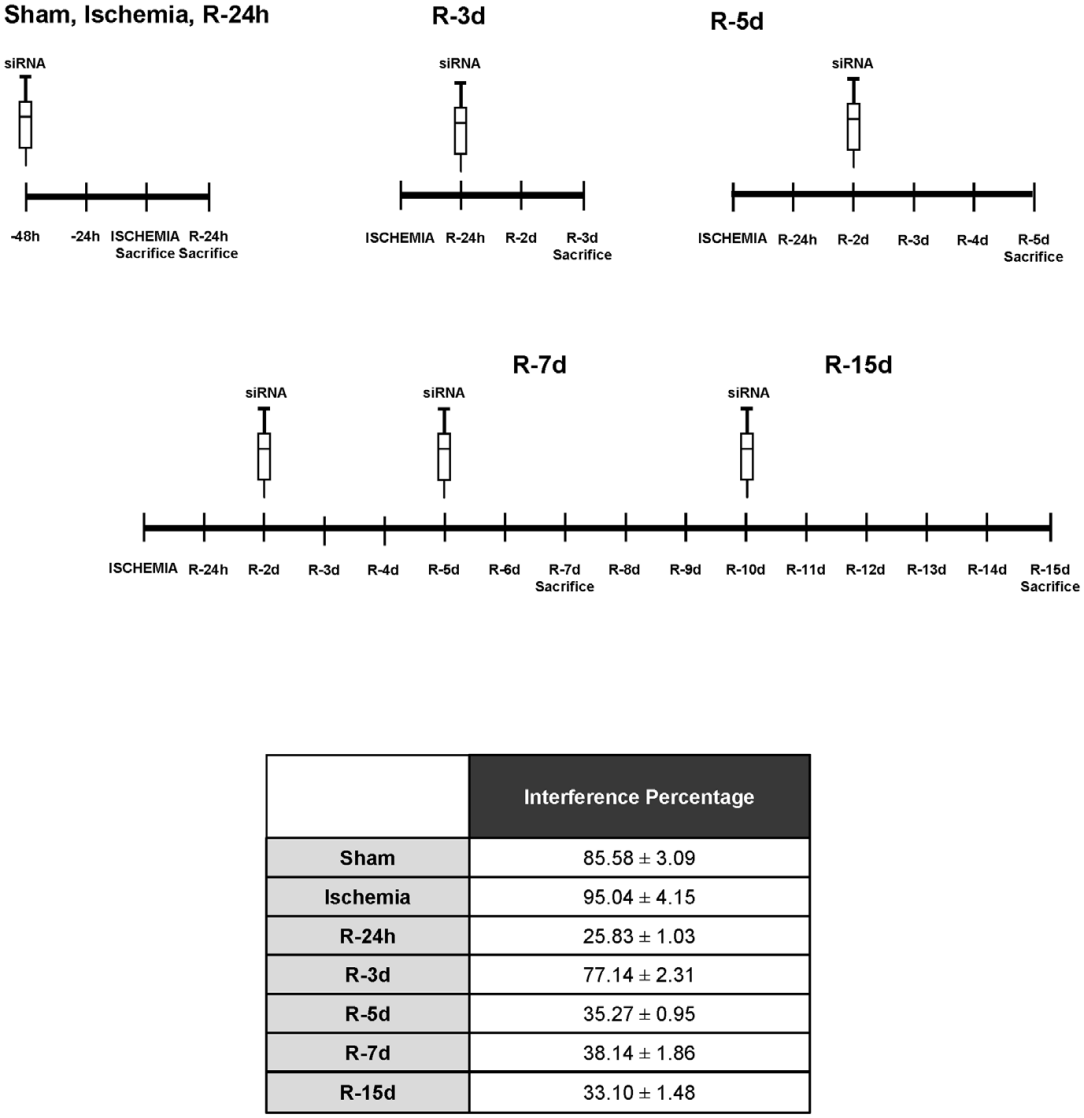
HIF-1α interference *in vivo*. (a) Scheme of siRNA and scramble treatments *in vivo.* SD Rats were injected with 100 nM of specific siRNA against HIF-1α or scramble through the tail vein at indicated times. (b) Percentage of HIF-1α interference estimated by qRT-PCR in total renal lysates from rats treated with HIF-1 α siRNA in comparison to rats treated with scramble in each condition.

**Figure 8:**
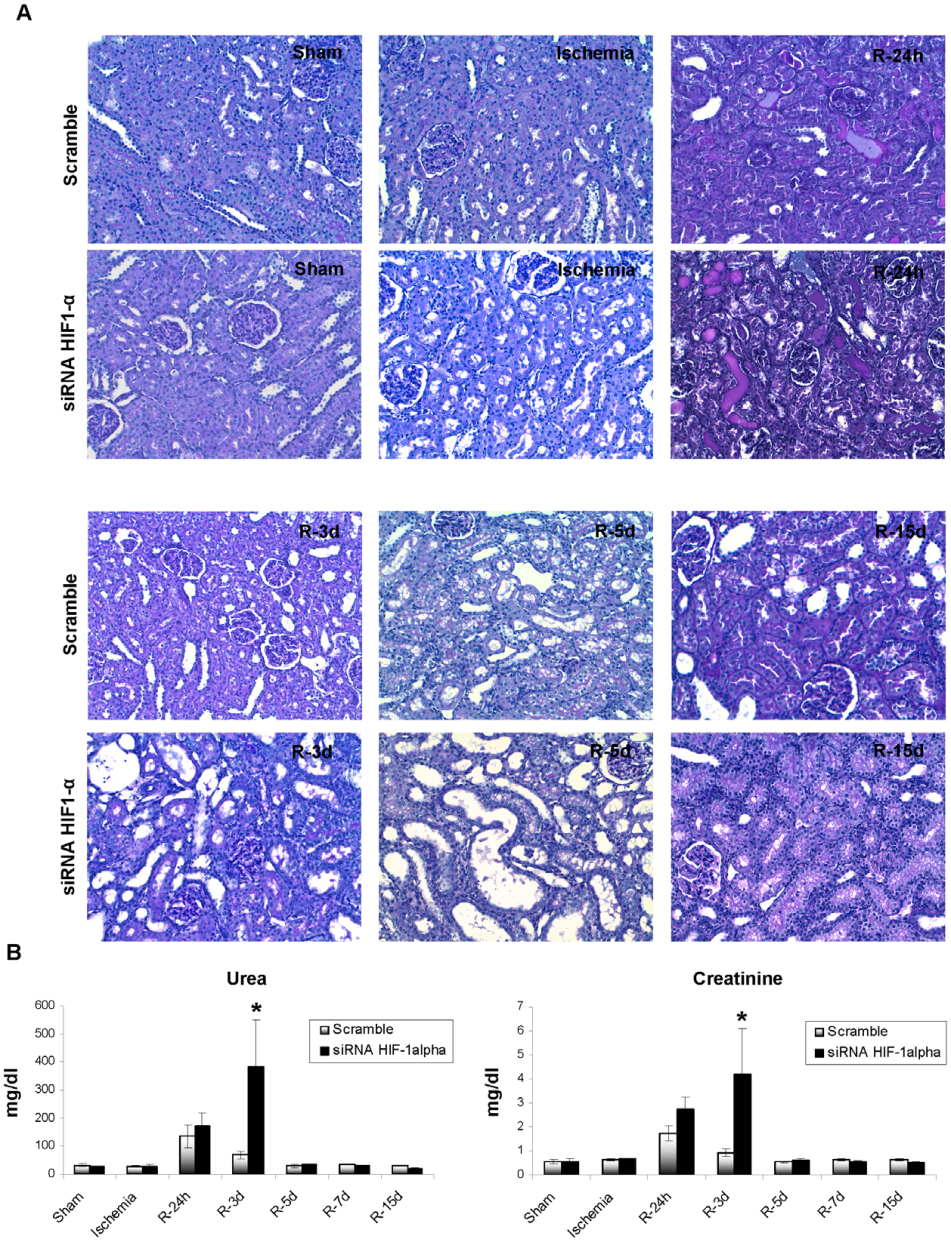
HIF-1α interference *in vivo* exacerbates I/R-induced renal injury. (a) PAS staining in paraffin-embedded renal tissue sections from SD rats during I/R, injected with scramble or with specific siRNA against HIF-1α. Note increased renal damage at 3 and 5 days of reperfusion in siRNA treated rats. Representative images are shown. Magnification: ×200. (b) Renal function estimated by serum creatinine and urea levels. Statistical significance was found compared to sham scramble condition, p≤0.05.

Next, the I/R model was used to elucidate the role of HIF-1 α during I/R *in vivo* by means of HIF-1 α interference. The protocol and interference efficiency *in vivo* are shown by measuring HIF-1 α mRNA levels in total renal lysates in figure 7. HIF-1 α interference in rats and analysis of renal histology by PAS staining and renal function by urea and creatinine levels in serum are shown in figure 8A and 8B.

HIF-1 α interference during reperfusion dramatically increases proximal tubule damage after 3 to 5 days, compared to scramble. Tubular alterations, mainly dilation and epithelial denudation are more evident in the interfered animals. Accordingly, renal function is worsened in interfered animals at 3 days of reperfusion. However, renal function is almost similar between scramble and interfered conditions later during reperfusion (5 days), correlating with the well known dichotomy between recovery of renal structure and creatinine/urea levels in this model.

These results demonstrate that HIF-1 α induction during reperfusion is an essential requirement for proximal tubule cell survival and consequently earlier recovery after renal ischemia.

### HIF-1 α expression in proximal tubules of human renal allograft correlates with less extended ATN

Our data from the experimental *in vitro* and *in vivo* models strongly suggested that HIF-1 α has a protective role against ischemic injury. Therefore we have used post-transplant human biopsies were ischemic damage is observed to confirm this observation.

Thus, we have assessed HIF-1 α expression in a set of 15 human renal biopsies with different ischemic ATN severity, obtained mostly between days 7 to 15 after transplantation. The characteristics of the biopsies are presented in table 1.

**Table 1:** Human postransplant Renal Biopsies features.

Biopsy ID	Recipient Gender	Recipient Age	Date of Transplantation	Type of Transplantation	Date of Biopsy (Days)	Indication of biopsy	Immunosupression treatment at biopsy
**1**	Female	50	22/05/08	Cadaveric	7	Graft dysfunction	Steroids/Basiliximab/Tacrolimus/MMF
**2**	Male	53	23/5/08	Cadaveric	15	Graft dysfunction	Tacrolimus
**3**	Male	62	21/01/08	Cadaveric	11	Graft dysfunction	Steroids/Tacrolimus/MMF
4**	Male	25	31/01/08	Cadaveric	12	Graft dysfunction	Steroids/Basiliximab/Tacrolimus/MMF
**5**	Female	48	22/01/08	Cadaveric	8	Graft Dysfuncion	Steroids/Tacrolimus/MMF
6**	Male	23	14/6/08	Cadaveric	24	Graft Dysfuncion	Steroids/CyA/MMF/Basiliximab
**7**	Male	73	21/6/07	Cadaveric	10	Graft Dysfuncion	Steroids/Tacrolimus/MMF/Basiliximab
**8**	Male	67	23/4/08	Cadaveric	9	Graft Dysfuncion	Steroids/Tacrolimus/Basiliximab
**9**	Male	62	28/1/08	Cadaveric	11	Graft Dysfuncion	Steroids/Tacrolimus/MMF/Basiliximab
**10**	Male	29	5/4/07	Cadaveric	7	Graft Dysfuncion	Steroids/Tacrolimus/MMF/Basiliximab
**11**	Male	53	7/7/06	Cadaveric	25	Graft Dysfuncion	Steroids/CyA/MMF/Basiliximab
**12**	Male	43	22/3/08	Cadaveric	5	Graft Dysfuncion	Steroids/Tacrolimus/MMF/Basiliximab
**13**	Male	23	14/6/08	Cadaveric	10	Graft Dysfuncion	Steroids/Tacrolimus/MMF/Basiliximab
14**	Male	73	21/6/07	Cadaveric	20	Graft Dysfuncion	Steroids/Tacrolimus/MMF/Basiliximab
**15**	Male	52	13/5/08	Cadaveric	8	Graft Dysfuncion	Steroids/Tacrolimus/MMF/Basiliximab

**Figure 9:**
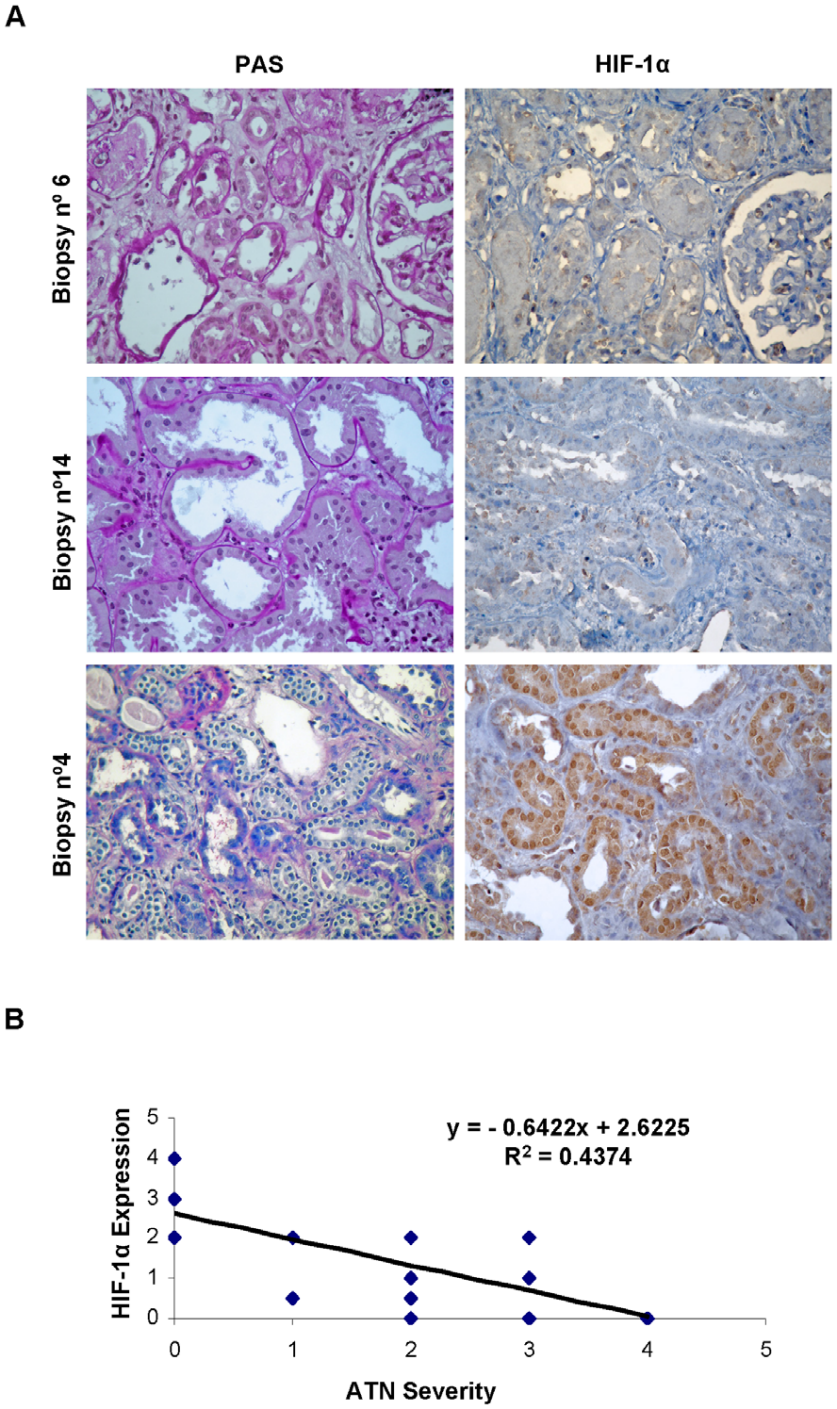
HIF-1α is expressed exclusively in non-damaged proximal tubules of human post-transplant renal biopsies. (a) PAS staining for renal structure and immunohistochemistry for HIF-1α in paraffin-embedded human renal biopsies. HIF-1 α is expressed in non-damaged proximal tubules (biopsy n°4). Images of representative biopsies are presented: severe ATN (biopsies n°6 and n°14) and ATN regeneration (biopsy n°4). Magnification: ×400. (b) Spearman Rho-Correlation coefficient between ATN grade and HIF-1α expression in all biopsies, with statistical significance p≤0.01.

ATN: acute tubular necrosis estimation; CyA: cyclosporine A; MMF: mophetil-mycophenolate. Biopsies which representative images are shown in figure 9 are marked with asterisks.

Representative images of PAS staining and HIF-1 α immunostaining are presented in figure 9A. Biopsies number 6 and 14 exhibited extensive ATN development with marked alterations in proximal tubules: loss of microvilli, loss of epithelium integrity and tubule denudation. Both expressed HIF-1 α. Biopsy number 4 exhibits less ATN with normal epithelium structure and morphology, presence of microvilli and proximal cell proliferation (for repair of denudated tubules). Interestingly, this biopsy showed strong HIF-1 α expression. ATN development vs recovery estimation following histopathological criteria as well as quantification of HIF-1 α expression in the biopsies is shown in table 2. Additionally, significant negative correlation by Rho Spearman analysis was found between HIF expression and ATN severity (figure 9B).

**Table 2:** Quantification of ATN development vs recovery HIF-1α expression in renal biopsies, by PAS staining and immunohistochemistry detection.

Biopsy ID	ATN/RECOVERY	HIF-1 α EXPRESSION
**1**	−/−	++
**2**	+/++	++
**3**	++/+	++
4**	−/+++	++++
**5**	+++/+	++
6**	++++/+	−
**7**	++/+	+/−
**8**	+++/++	+
**9**	++/+	++
**10**	−/++	+++
**11**	++/++	++
**12**	+++/−	−
**13**	++/−	−
14**	+/++	+/−
**15**	++/+	+

Biopsies which representative images are shown in figure 9 are marked with asterisks.

All these findings demonstrate that HIF-1 α is expressed during the post-transplant period in proximal tubules which exhibit minor ATN and recovered morphology, strongly suggesting that HIF-1 α stabilized during reperfusion is required for proximal tubule survival and repair after ischemic damage.

## Discussion

We demonstrate that HIF-1 α is accumulated after renal ischemia and unexpectedly, also in reperfusion. This induction is observed in human proximal tubule cells after H/R, in rat kidneys after I/R and in human biopsies post-transplant. HIF-1 α induction during reperfusion is crucial for proximal tubule epithelial cell survival and recovery after I/R as the interference approaches *in vitro* and *in vivo* demonstrated. Indeed, HIF-1 α drives target genes expression, including genes involved in tissue repair such as EPO or VEGF. Our data in human biopsies strongly suggested that HIF-1 α is essential in ATN recovery and might play a critical role in allograft outcome. *In vivo* and *in vitro* approaches demonstrated that this HIF-1 α accumulation is not related to low oxygen tension, but rather to Akt/mTOR pathway activation.

HIF-1 α induction during renal ischemia has been extensively studied [16]. However, HIF-1 α induction during *in vivo* reperfusion has not been previously described although stabilization of this factor during reoxygenation in endothelial cells has been reported [17]. In humans, HIF-1 α expression in renal allograft several weeks after transplantation was suggested to be probably related to a chronic hypoxia caused by hyperfiltration, hyperthrophy, calcineurin inhibitor-induced toxicity or combination of them [18]. Our results using pimonidazole in rats strongly support that HIF-1 α induction during reperfusion is not the consequence of low oxygen tension in the renal parenchyma. Accordingly, using an *in vitro* model which mimics the proximal tubule damage and repair induced by I/R *in vivo* [12] we demonstrate that HIF-1 α is induced also during the reoxygenation period. Moreover, an *in vitro* protocol of depletion/replenishment of nutrients, where oxygen tension is not modified, also reproduced the second HIF-1 α induction, confirming that HIF-1 α is also induced during reperfusion/reoxygenation in an O_2_-independent manner.

HIF-1 α protein is significantly accumulated during reperfusion as the result of protein synthesis, since mRNA levels did not change significantly. HIF-1 α can be detected in normoxia in response to growth factors, hormones or cytokines [19]. Activation of PI3K/Akt/mTOR signalling pathways leading to increase in protein translation has been described as the main mechanism of normoxic-HIF-1 α accumulation [5]. Our findings demonstrate that Akt is activated early during reoxygenation followed by HIF-1 α induction. Moreover, LY 292004 and rapamycin prevents HIF-1 α induction during reoxygenation, suggesting that, in our system, the Akt/mTOR pathway might affect HIF-1 α levels by promoting protein translation as previously described [20]. We also observed Akt activation in the *in vitro* protocol of D/R, correlating with HIF-1 α protein induction. Moreover, previous works of our laboratory demonstrated that Akt is activated during reperfusion in our model of I/R [11].

Akt activation by I/R in several organs including kidney has been already reported [21]. *In vitro*, in a model of H/R which did not include nutrient alterations Kwon et al., 2006 [22] described PI3K/Akt and ERK1/2 activation during reoxygenation. In our H/R protocol, Akt is probably activated through growth factors and might involve Ras, which is also activated in our H/R protocol, as previous works of our laboratory reported [23]. It is well established that Ras activation might lead to p70S6K phosphorylation through ERK, contributing also to promote translation [20]. On the other hand, it has been recently reported that metabolic changes underlying cell adaptation to deprivation/replenishement of nutrients can lead also to HIF-1 α stabilization, by specific inhibition of PHDs [24]. Thus, alterations in metabolic intermediaries could also contribute to the normoxic HIF-1 α induction reported here.

Outstandingly, in human biopsies, HIF-1 α was exclusively detected in proximal tubule cells which did not exhibit marked ischemic damage or show regeneration features. Moreover, the strongest expression of HIF-1 α was observed in the biopsies which clearly exhibited tubule regeneration (biopsy n°4). Additionally, in the *in vivo* I/R rat model, HIF-1 α is induced during reperfusion when tubule repair is taking place and renal function is being restored [25], [9], strongly suggesting that HIF-1 α mediates proximal cell survival/regeneration during I/R. Proving this hypothesis, our *in vitro* findings show that both HIF-1 α inductions are critical for HK2 cells survival during H/R. Indeed, inhibition of both HIF-1 α inductions by specific siRNA or YC-1, lead to cell death. Notably, neither siRNA nor YC-1 affects Akt activation during reoxygenation.

HIF-1 α mediates proximal tubule cell survival in response to hypoxia as the result of cell adaptation to low oxygen tension. Indeed, promotion of HIF-1 α previous to hypoxia/ischemia in *in vivo* models inhibited proximal tubule cell death and reduced renal damage [10]. By contrast, the lack of HIF-1 α stabilization promoted cell death after ischemia [9], [26]. Hypoxia-induced HIF-1 α is also at the base of the beneficial effects of ischemic preconditioning [27]. Until now, no research has considered HIF-1 α induction during reperfusion as a crucial factor for proximal epithelial cell regeneration during I/R, which our results strongly suggest.

Both HIF-1 α protein inductions lead to target gene expression as luciferase assays showed which most probably mediates HIF-1 α effects. PHD3 induction was the most prominent induced gene during hypoxia as previously demonstrated [28]. Both PHD3 and VEGF remained elevated during reoxygenation might be due to second HIF-1 α induction, as the D/R protocol suggested. Maintained PHD3 levels until 3–6 h of reoxygenation could contribute to further degradation of the normoxic HIF-1 α stabilization. Notably, EPO mRNA expression showed a robust induction at 3 h of reoxygenation suggesting a main regulation of this gene during reperfusion. Thus it is conceivable that the two HIF-1 α inductions might result in differential gene expression related to different roles of HIF-1 α in the proximal tubule cell response to I/R. Indeed, the first HIF-1 α induction might control the cell adaptation response to hypoxia and the second induction during reperfusion might mediate proximal tubule repair.

The HIF-1 α target genes EPO and VEGF, which were markedly expressed during reoxygenation *in vitro,* would be involved in proximal tubule regeneration *in vivo* as previously suggested [29], [30], [31]. This hypothesis is supported by our qRT-PCR findings in rats. Related to this, we demonstrated a significant negative correlation between HIF-1 α expression and ATN development. Accordingly, it was proposed that the expression of HIF-1 α in human allograft biopsies could contribute to ameliorate renal damage associated with re-flow and improve renal allograft outcome [18]. On the other hand, it is important to note that the long sustained expression of HIF-1 α and VEGF has been also related with immunological renal allograft rejection as well as fibrosis development [32]. Therefore, further studies on long term human allograft biopsies are required to assess this issue.

Remarkably in our work and consistent with the human biopsies, interference of reperfusion-induced HIF-1 α in rats aggravates renal damage in structure and function, demonstrating that this normoxic HIF-1 α induction by itself is crucial for renal outcome after I/R. Although creatinine and urea return to normal levels in both scramble and siRNA rats, kidney structure is impaired for longer in interfered rats. It is well known that even if creatinine and urea normalize, renal function, in terms of ions re-absorption and volume regulation could be still affected [33], [34]. Thus, HIF-1 α induction during reperfusion would be a requirement for renal structure and function recovery after ischemic damage. In this regard, our laboratory recently demonstrated that BN rats which recover faster from I/R-induced renal damage, present higher levels of HIF-1 α and some of its target genes [11]. Moreover, current studies of our lab indicate that HIF-1α could contribute to renal tissue repair after I/R by regulating the inflammatory response, including IL-1β expression, among other mechanisms.

In summary, we are reporting here a novel HIF-1 α induction in reperfusion after renal ischemia. This accumulation is critical for epithelial cell survival and repair, promoting tissue repair genes expression. Interventions based on HIF-1 α during reperfusion in the post-transplant period could be an efficient therapy strategy to improve renal allograft outcome.

## Materials and Methods

### Renal biopsies collection: Ethics statment and immunohistochemistry

This study was approved by the ethics committees of Hospital Universitario Ramón y Cajal and Hospital La Paz. Tissue extraction was performed as standard renal biopsy at Nephrology Departments, after patient written approval and according to the Spanish and European legislation. From 60 post-transplantation biopsies that were performed between day 7 and day 15 due to renal dysfunction, 15 anonymized biopsies showing ATN of ischemic ethiology, as the only diagnosis, were selected for this study. Samples were fixed in formaldehyde, included in paraffin and 4 µm slides were used for periodic acid-Schiff staining or for immunohistochemistry. For biopsies, histopathologic evaluation was performed in a blinded fashion by two independent observers. Tissue sections were rated from (−) to (++++), scoring morphological alterations in proximal tubule cells, brush border loss, detached and necrotic cells in proximal tubules and presence of intraluminal casts and infiltrating cells.

Immunostaining was performed as previously described [23], using as primary antibodies anti-HIF-1 α (Santa Cruz) 1∶25. Slides were observed with Nikon Eclipse 200T microscopy.

### I/R model in rat: Ethics Statement. Pimonidazole treatment and immunohistochemistry

Male Sprague-Dawley rats (180–200 g) from our own colony were divided in groups of 5–7 animals for each condition. Animals were treated according to the Spanish guidelines (RD 1201/2005) that are in compliance with the EU Guide for the Care and Use of Laboratory Animals. The experimental protocol was approved by the Internal Committee for Animal Ethics of Hospital Universitario Ramón y Cajal. Rats were anesthetized with an inhaled anesthesia mixture of 2% isoflurane (Abbott Laboratories Ltd., Queenborough, Kent, England) and 1 l/min oxygen and placed on a temperature-regulated table (37°C). Renal ischemia was performed by clamping both renal pedicles during 45 min. Sham-operated group underwent the same surgical procedure without clamping. Animals were sacrificed at different times of reperfusion and kidneys were harvested.

In the case of ischemia condition, 60 mg/Kg of pimonidazole (Hydroxyprobe ™-1) was injected intraperitonealy 60 minutes before clamping and animal were sacrificed after ischemia. In the case of 5 days of reperfusion (R-5d), the same pimonidazole concentration was injected 60 minutes before sacrifice.

Immunohistochemistry for HIF-1 α in kidney rat sections was performed as described above for human biopsies. For pimonidazole staining Hydroxyprobe ™-1 Mab1 (monoclonal antibody HRP conjugated) at 1∶50 was used.

### Cell culture, H/R and D/R *in vitro* protocol. Cell treatments

HK-2 cells (ATCC) were cultured in DMEM/F12 containing 10% FBS, 1 g/l insulin, 0.55 g/l transferrin, 0.67 mg/l selenium (Invitrogen), 2 mM glutamine, 100 U/ml penicillin and 100 µg/ml streptomycin (Invitrogen), in a humidified atmosphere with 5% CO2 at 37°C. Cells were cultured until confluence and then they were serum deprived for 24 hours. Monolayers were cultured for 6 hours in HBSS (Invitrogen), in a hypoxic atmosphere containing 1% O_2_, 94% N_2_, 5% CO_2_ (Air Liquide). After hypoxia, cells were maintained in complete medium and 21% O_2_ for reoxygenation [12]. Serum-starved cells following 6 h in HBSS were used as Control. For depletion/replenishment (D/R) protocol, cells were subjected to all changes of media described above, but maintaining standard 21% O_2_ (Figure 2A).

We used 10 µM YC-1 (3-(5′-hydroxymethyl-2′-furyl)-1-benzylindazole) (Alexis Corporation), for HIF-1 α inhibition, 50 µM LY 294002 (Calbiochem) for Akt inhibition and 20 nM rapamycin (Calbiochem) for mTOR inhibition.

### Western blotting: Tissue and cell lysates

Western blotting in tissue and cells were performed as previously described [11]. We used 1/250 for anti-human anti-HIF-1 α (BD Transduction Laboratories); 1/1000 for p-AKT (Cell Signalling), for total Akt (Cell Signalling) and for total actin (Santa Cruz); 1/500 for p-S6Kp70 (Cell Signalling).

### HIF-1 α siRNA transfection *in vitro* and *in vivo*

HK2 at 70% of confluence were transfected with 100 nM of 4 different HIF-1 α siRNAs (Customized Silencer Select siRNAs: S225214, S225215, S225216 Ambion; siRNA sc-44225, Santa Cruz Biotechnologies) and scramble siRNA (sc-37007, Sta Cruz Biotechnologies), using Lipofectamine 2000 according to the manufacturer's protocol. Transfected cells were subjected to H/R after 48 h of transfection. siRNA sc-44225 (Sta. Cruz) was selected for the *in vitro* experiments due to its high efficiency.

*In vivo*, siRNA sc-44225 containing 3 sequences against 3 different HIF-1 α exons, was used for HIF-1 α inhibition. 75 µg/kg body weight of siRNA mixed with Jetpei (Polyplus, Genycell) following manufacturer's instructions was injected to animals, by the tail vein, 48 h or 72 h before sacrifice, as detailed in figure 7.

### Cell death quantification

For each sample supernatants containing detached and death cells were collected and attached cells were trypsinized. Cell suspensions were stained with 50 µg/ml propidium iodide (PI) in PBS containing 0.1% NP40.

Cell cycle distribution was determined using a Beckton Dickinson FACScan flow cytometer, analysing 20.000 cells per sample. The percentage of cells in sub-G_1_ phase (apoptotic cells) was estimated using Modfit 2.0 software (Beckton Dickinson).

### Quantitative RT-PCR

Cells or renal tissue were lysed into 1 ml of Tri-Reagent (Ambion). Total RNA was extracted and quantified. cDNA was obtained from 1 µg of total RNA from each sample (Improm-II reverse transcriptase, Promega) and 1 µl of cDNA sample was used as template for PCR with LC Fast Start DNA master SYBR Green I kit (Roche Applied Science) following the manufacturer's instructions. PCR were carried out in Lightcycler 480 equipment (Roche). For each sample and experiment, triplicates were made and normalized by 28S mRNA levels. Primer pairs used are shown in table 3.

**Table 3:** Primer sequences used for qRT-PCR *in vivo* and *in vitro.*

	Forward 5′→3′	Reverse 5′→3′
**28 s**	CAG TAC GAA TAC AGA CCG	GGC AAC AAC ACA TCA TCA G
**HIF-1α**	GTT TAC TAA AGG ACA AGT CACC	TTC TGT TTG TTG AAG GGA G
**EPO**	GCA TGT GGA TAA AGC GCT C	CCC GGA GGA AAT TGG AGT AG
**VEGF**	TGC CAA GTG GTC CCA G	GTG AGG TCT TGA TCC G
**PHD3**	GAT GCT GAA GAA AGG GC	CTG GCA AAG AGA GTA TCT G

### Luciferase assays

HK-2 cells seeded on 24-well plates were transiently transfected with 9xHRE-luc-PGLN3 (prolactin minimum promoter) [15] (800 ng/well) and renillaluc-pSRLV40 (0,4 ng/well) using Lipofectamine 2000 (Invitrogen). After 24 h of transfection, cells were subjected to H/R. Cells were lysed and luciferase activity was estimated using Promega Dual Luciferase Kit (Promega). Results were expressed as the ratio firefly luciferase/renilla luciferase counts. All data are expressed as mean ± SEM.

### Statistical analysis

Data are presented as mean ± SEM. After the Levene test of homogeneity of variance, the Kruskal-Wallis test was used for group comparison. A p<0.05 was considered significant. In case of significant differences, intergroup differences were analyzed by post-hoc Mann-Whitney U tests with the Bonferroni correction. Spearman Rho correlation coefficient was used for analysis in human biopsies. Statistical analysis was carried out with Statistical Package for the Social Sciences (SPSS) version 15.0.
